# Hybrid Deep Learning for Medication-Related Information Extraction From Clinical Texts in French: MedExt Algorithm Development Study

**DOI:** 10.2196/17934

**Published:** 2021-03-16

**Authors:** Jordan Jouffroy, Sarah F Feldman, Ivan Lerner, Bastien Rance, Anita Burgun, Antoine Neuraz

**Affiliations:** 1 Department of Biomedical Informatics Necker-Enfants malades Hospital Assistance Publique–Hôpitaux de Paris Paris France; 2 UMRS 1138 team 22 Institut National de la Santé et de la Recherche Médicale Université de Paris Paris France; 3 Department of Biomedical Informatics Georges Pompidou European Hospital Assistance Publique–Hôpitaux de Paris Paris France

**Keywords:** medication information, natural language processing, electronic health records, deep learning, rule-based system, recurrent neural network, hybrid system

## Abstract

**Background:**

Information related to patient medication is crucial for health care; however, up to 80% of the information resides solely in unstructured text. Manual extraction is difficult and time-consuming, and there is not a lot of research on natural language processing extracting medical information from unstructured text from French corpora.

**Objective:**

We aimed to develop a system to extract medication-related information from clinical text written in French.

**Methods:**

We developed a hybrid system combining an expert rule–based system, contextual word embedding (embedding for language model) trained on clinical notes, and a deep recurrent neural network (bidirectional long short term memory–conditional random field). The task consisted of extracting drug mentions and their related information (eg, dosage, frequency, duration, route, condition). We manually annotated 320 clinical notes from a French clinical data warehouse to train and evaluate the model. We compared the performance of our approach to those of standard approaches: rule-based or machine learning only and classic word embeddings. We evaluated the models using token-level recall, precision, and F-measure.

**Results:**

The overall F-measure was 89.9% (precision 90.8; recall: 89.2) when combining expert rules and contextualized embeddings, compared to 88.1% (precision 89.5; recall 87.2) without expert rules or contextualized embeddings. The F-measures for each category were 95.3% for medication name, 64.4% for drug class mentions, 95.3% for dosage, 92.2% for frequency, 78.8% for duration, and 62.2% for condition of the intake.

**Conclusions:**

Associating expert rules, deep contextualized embedding, and deep neural networks improved medication information extraction. Our results revealed a synergy when associating expert knowledge and latent knowledge.

## Introduction

In 2017, medication consumption in France represented €37.8 billion (approximately US $45.5 billion) in spending and 16% of the French health budget [1]. Adverse drug reactions are an important public health problem, representing a major cause of mortality (0.15% in France); one-third of admissions caused by adverse drug reactions are preventable, associated with a poorly reported drug history or rare adverse events [2,3].

Furthermore, electronic health records contain rich information about drug history that would be valuable to the care of patients (eg, to prevent interaction with another medication and to track side effects), for epidemiology, or pharmaco-vigilance [4]. A major hurdle in the use of electronic health records is the format of the data. Up to 80% of relevant clinical information is present solely in the form of unstructured text, which represents a major barrier to the secondary use of this type of information [5,6].

To overcome this issue, natural language processing techniques can be used to extract, normalize, and restructure drug-related information from clinical texts [6,7] and increase the information available for research and health care. Three approaches have been described for this task: expert knowledge modeling, machine learning, and hybrid methods (combining both).

The first approach relies on modeling expert knowledge using dictionaries or rules (ie, expert rules) such as MedEx, MedXN, or MedLEE based on lexicons or regular expressions [8-12]. Dictionary-based approaches allow for direct or approximate matching of terms from a dictionary or terminology. These approaches may offer poor results when the mentions used in texts deviate from the terms in the dictionary. Rule-based approaches allow for specific extractions but usually lack sensitivity and do not perform well on new data sets. Rule-based approaches also require domain experts to design and build the rules and are particularly time-consuming. In addition, expertise is rare and costly, which constitutes a severe bottleneck for the use of this type of method.

The second approach, using machine learning, has been developed in addition to expert approaches to extract medication name, dosage, frequency, duration, mode, reason for the intake and to detect adverse drug reactions [13,14]. Most systems included a conditional random field or a support vector machine for medication-related information extraction [15-18], 2 studies introduced bidirectional long short-term memory associated with conditional random field for named entity recognition and medication information extraction [19,20], and another used a semisupervised model [21].

For the 2018 N2C2 shared task on medication extraction in electronic health records [22], several systems were proposed. The data set used in the challenge consisted of 505 discharge summaries extracted from the MIMIC-III database [23]. This data set contained 16,225 drug mentions in the training set and a total of 50,951 entity annotations again in the training set. Among the best-performing algorithms, bidirectional long short term memory and bidirectional long short term memory with conditional random field architectures were popular [24-27]. Some systems combined attention mechanisms [28] or convolutional neural networks [27]. Others combined classic entity extraction systems such as cTakes with classifiers such as support vector machines [29]. Ensemble approaches, combining multiple classifiers were also proposed [24-26,30].

At the conjunction of machine learning and expert rules, hybrid approaches can leverage the frugality of expert rules (in terms of data needs) and the flexibility and generalizability of machine learning. Examples include identifying medication heading using a conditional random field for named entity identification and a support vector machine to classify relations combined with a rule-based context engine [31]; a conditional random field and 2 bidirectional long short term memory–conditional random field models to extract handcrafted features [25]; and using expert rules and a knowledge base to enrich text, then using a bidirectional long short term memory with attention to perform the medication extraction in electronic health records [28]. These approaches were designed for text written in English. To the best of our knowledge, there are only a few studies [32,33] on French corpora: Deleger et al [32] used a rule-based system, and Lerner et al [33] developed a hybrid system that associated expert rules using terminology and bidirectional gated recurrent units with a conditional random field.

In recent years, the adoption of word embedding methods has led to a significant increase in the level of performance achievable by many natural language processing tasks [34]. Word embeddings use dense vector representation of the vocabulary. Interestingly, word embeddings are computed using large amounts of unannotated data (eg, Wikipedia). In static word embeddings, a token is represented by a static numeric vector. Recently, contextual word embedding methods have appeared, such as embedding for language model [35]. Contextual word embeddings provide a varying representation of the tokens with regard to the context in the text. Contextual word embeddings lead to richer representations and help to improve the performance in clinical concept extraction tasks [36]; results further improve when semantic information is incorporated [37].

In this work, we aimed to extract medication-related information from clinical narratives written in French in a real-world setting (ie, with documents directly extracted from a clinical data warehouse). Once extracted, such information can be restructured to be used for different purposes (eg, clinical epidemiology, monitoring, pharmaco-epidemiology, adverse drug reaction detection). Our purpose was two-fold: (1) We aimed to develop a gold standard data set of annotated clinical documents in French, along with an annotation guide, and (2) we aimed to develop a hybrid approach combining an association of knowledge base and expert rules, contextualized word embeddings training on clinical text, and a deep learning model based on bidirectional long short term memory–conditional random field.

## Methods

### Data

#### Source

We leveraged the clinical data warehouse of the *Assistance Publique–Hôpitaux de Paris* (AP-HP), grouping data collected from 39 hospitals to build a data set of 1 million documents [38]. These clinical reports were medical prescriptions, discharge reports, examinations, observation reports, and emergency visits randomly selected from the clinical data warehouse.

#### Annotated Data Set

We created an annotated data set for training and evaluation. We iteratively developed an annotation guide during the first phase of annotation. A small portion of the extracted data set (320 documents) was manually annotated by 3 medical doctors using an annotation tool [39]. The annotations were converted to the inside, outside, beginning (IOB) standard. Tokens that refer to an entity were labeled *B-entity_type* for the first token and then *I-entity_type*, tokens outside entities mention are labeled O. We split the 320 annotated clinical notes in a training set (n=216), a development set (n=24), and a test set (n=80).

#### Knowledge Base for Drug Names

We relied upon 2 French databases—*Base de données publique des medicaments* (a publicly accessible, *National drug database*)[40] and OpenMedic, a database from the national medical insurance agency [41]. These 2 databases contain all the drugs distributed in France during a given year. They were mapped to the Anatomical Therapeutic Chemical classification system. We extracted data from 2015 to 2019 and created a curated and unified dictionary of drug mentions.

The corpus can be made available on the condition that a research project is accepted by the scientific and ethics committee of the AP-HP health data warehouse.

**Figure 1:**
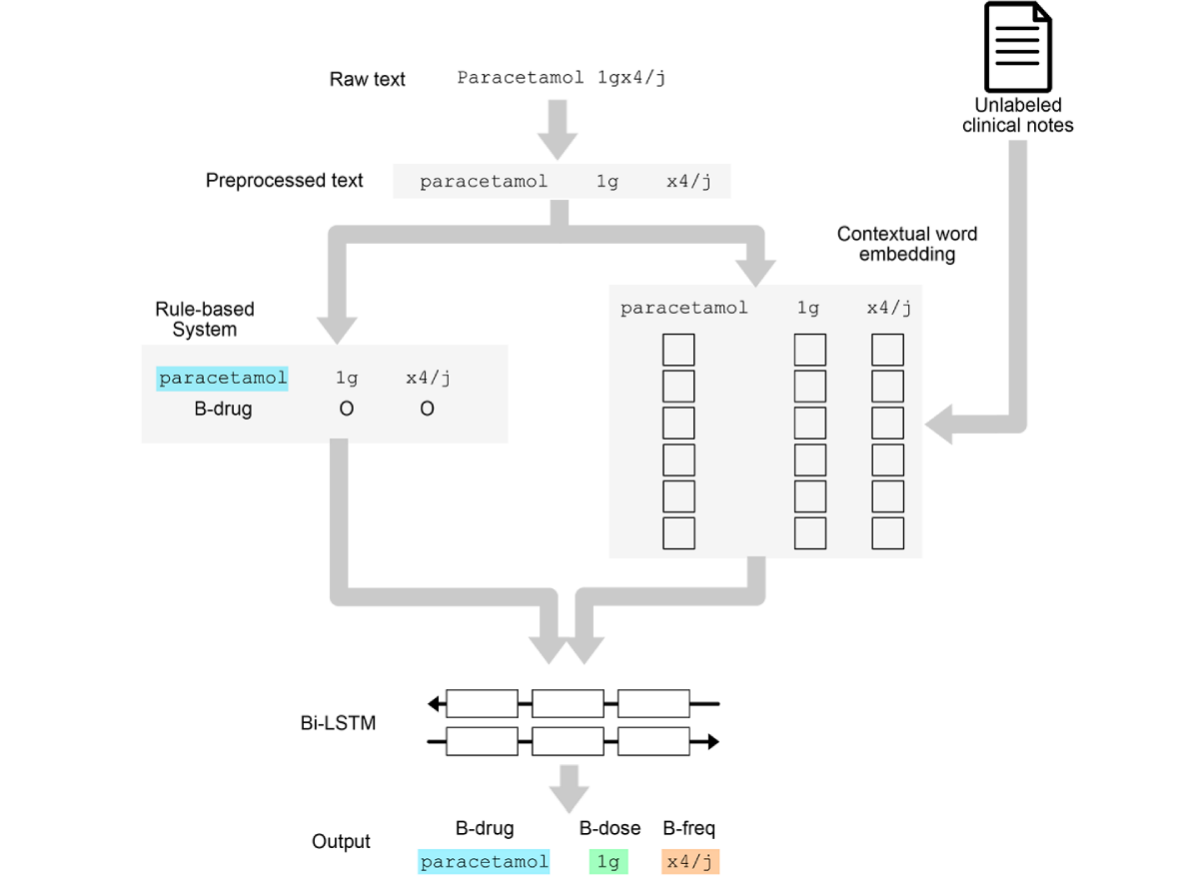
General architecture of the model. BiLSTM: bidirectional long short term memory; CRF: conditional random field.

**Figure 2:**
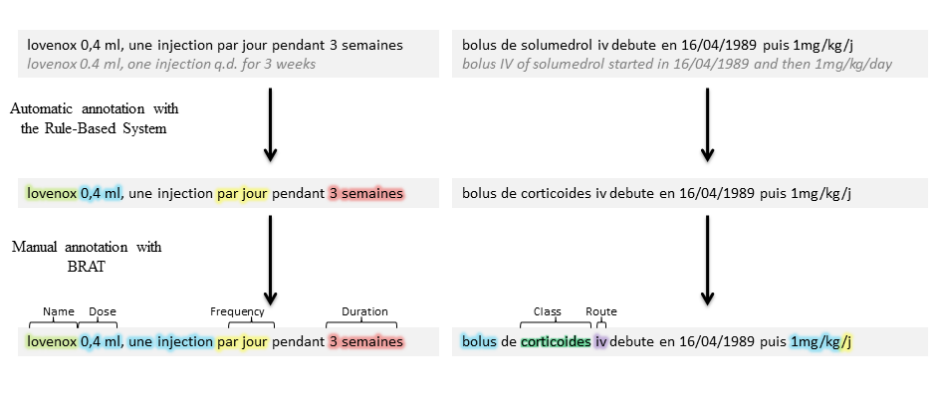
Annotation process with automatic annotation and completion with manual annotation.

After preprocessing, the text was preannotated using a set of expert handcrafted rules, then the texts were embedded using contextual word embeddings trained on a large corpus of clinical texts. The preannotations and the embedded texts were input into a bidirectional long short term memory–conditional random field to produce the final annotations (Figure 1; Figure 2).

### Task Definition

We aimed to identify medication-related information in clinical documents in French. We were interested in drug names and a set of attributes related to the drug mentions: dosage, frequency, duration, route, and condition of administration. A detailed description of the types of entities is provided in Table 1.

**Table 1:** Description of the task.

Type	Description	Examples
Medication name	Descriptions that denote any medication, active molecule, association or protocol	doliprane, paracetamol, augmentin
Medication class	Descriptions that denote any Anatomical Therapeutic Chemical class or common therapy	ß-Lactam, antibiotherapy
Dosage	Dose or concentration of medication in prescription	3 mg, 2 tablets
Frequency	Frequency of medication administration	3 per day, every morning
Duration	Time range for the administration	3 weeks, until the surgery
Route	Medication administration mode	intravenous, per os
Condition	The event which provokes the administration	if pain, if infection

### Preprocessing

We preprocessed the input texts as described in Textbox 1.

**Text preprocessing.**

Steps

- Removing acronym points and replacing decimal points by comma
- Removing break lines added during documents conversion to text
- Removing accents
- Replacing apostrophes by spaces
- Detecting sentence boundaries: remaining points or break lines without transitive verbs, preposition or coordinating conjunctions.
- Detecting word boundaries and tokenization: sequence of alphanumeric characters or a repetition of a unique nonalphanumeric characters

### Rule-Based Module

The overall approach was organized as follows: we first identified a drug mention or a drug-class mention with the knowledge-based dictionary using exact matching. The choice of exact matching for this step was driven by maximizing the precision of the annotations in this preannotation step. Then, using the identified mention as an anchor, we extended the search to the attributes of this mention (ie, frequency, dosage, duration, mode of administration, and condition of administration) in the area surrounding the seed mention. The attributes were detected using a set of handcrafted rules using regular expressions. Examples of the rules are described in Table S1 of Multimedia Appendix 1. At this stage, the annotated entities were identified by their position and length relative to the beginning of the document. For the next steps, the annotations were converted to the IOB standard. The output of the rule-based system was used for preannotating the documents before the manual annotation step to speed up the annotation process of the gold standard data set and to serve as extra features to the input of the deep-learning module.

### Deep Learning Module

#### Overview

We designed an approach leveraging deep neural networks. We tested 3 types of word embeddings—skip-gram [42], FastText embeddings [43], and embedding for language model [35]—and 2 neural network architectures—bidirectional long short term memory and bidirectional long short term memory–conditional random field.

#### Embeddings

We evaluated the impact of the word embeddings on the performance of the model. Our baseline was created using a skip-gram embedding trained on the training set only. We also considered FastText embedding (skip-gram model augmented with sub–word information) trained on a corpus of 1 million documents. Finally, we used embedding for language model embeddings, trained on 100,000 clinical notes that were contextualized embeddings computed through the internal states of a large bidirectional language model. The embeddings were kept fixed during model training.

#### Combination of the Rule-Based System Output

The output of the rule-based system was converted to the IOB standard. Then, this information was added as features to the input of the deep-learning module by concatenation with the word-embedding vectors.

#### Models

We used a deep recurrent neural network composed of long short-term memory units [44]. Specifically, we used bidirectional long short term memory composed of 2 concatenated long short term memory layers—one reading the input sequence forward, and another one reading the input sequence backward—allowing the model to take advantage of the context on the left and the right of a token when computing the latent states. The final prediction layer was either a standard dense layer with softmax or a conditional random field such as that in [19].

### Implementation and Optimization of Hyperparameters

We implemented all the models using Keras and Keras-contrib [45] libraries using Python (version 3) with a TensorFlow backend [46]. We trained our models for 50 epochs, using an ADAM optimizer [47] with a learning rate of 0.001 and early stopping with a patience of 8 epochs. We applied a decrease of learning rate on plateau using a factor of 0.1. For models with a final dense layer, we used categorical cross-entropy loss and softmax activation. For the models with conditional random field, we used marginal optimization and categorical cross-entropy loss. We tuned (using Hyperas version 0.4) the following hyperparameters using a random search with 15 iterations on the parameter space: batch size: 64, 128; long short term memory size: 128, 256, 512; dropout before and after long short term memory; and recurrent dropout: 0.0, 0.1, 0.2, 0.3, 0.5, 0.6, 0.7 (Table S2, Multimedia Appendix 1). All models were trained using NVIDIA P40 GPUs (3840 CUDA cores, 24 GB of DDRAM).

### Evaluation

#### Models

We compared the performance of the rule-based system only, bidirectional long short term memory only, and rule-based system plus bidirectional long short term memory (with and without conditional random field). For bidirectional long short term memory with and without conditional random field models, we tested the impact of adding FastText embeddings or embedding for language model embeddings.

#### Metrics

We considered an extracted token to be a true positive if it was annotated with the correct category, a false positive if it was falsely annotated with respect to the evaluated class, and a false negative if it was not annotated or if it was annotated with an incorrect class. We computed the precision, recall, and F-measure to evaluate each model, microaveraging over all entries (Multimedia Appendix 2)

We also used the slot error rate metric. A slot corresponded to a mention of an entity (ie, a sequence of B and I tokens of the same class), a deletion was a missing slot, an addition was a slot that had been incorrectly added, a substitution or type error was a class that had been replaced by another class, and a frontier error was a token that had been added or removed at the end or the start of the slot [48].

## Results

### Annotated Data Set

The labeled data set contained 320 clinical notes and 19,957 sentences with 173,796 words. Training, development, and test sets included 216, 24, and 80 clinical notes with 13,737, 1373, and 4847 sentences, respectively. Table 2 summarizes the number of tokens and slots for each class in each data set.

**Table 2:** Number of slots and tokens for each class per data set.

Label	Train		Development			Test	
	Tokens	Slots	Tokens	Slots	Tokens		Slots
Medication name	1385	1227	146	143	450		398
Medication class	309	228	38	30	97		76
Dosage	1366	761	115	62	606		311
Frequency	1604	600	142	46	468		184
Duration	161	70	26	13	68		37
Route	95	85	8	8	69		55
Condition	192	61	9	3	89		28

### Overall Comparison of the Models

Table 3 summarizes the results of the different models. Overall, the best models were the hybrid models combining rule-based system, text embedding with embedding for language model, and bidirectional long short term memory (F-measure: 89.86). It had the lowest slot error rate (0.19) with a minimal deletion rate (0.05).

The bidirectional long short term memory with baseline embedding had the worst results (F-measure: 73.93). Adding FastText and embedding for language model trained on external data sets increased the F-measure by 14.15 and 9.81 points respectively. Combining rule-based system and bidirectional long short term memory increased the F-measure by 14.1 points.

The rule-based system alone had the highest precision (94.67) with the lowest insertion (0.03) and frontier (0.04) error rates. It had the second-lowest type error rate (0.02) but one of the highest deletion error rates (0.23). Adding bidirectional long short term memory and embedding for language model to the rule-based system increased the F-measure by 10.45 points.

**Table 3:** Overall medication component information predictions metrics by models.

Model^a^	F-measure	Precision	Recall	Slot error rate	Insertion error rate	Deletion error rate	Type error rate	Frontier error rate
RBS^b^	79.41	94.67	72.28	0.29	0.03	0.23	0.02	0.04
BiLSTM^c^	73.93	83.89	67.57	0.45	0.09	0.25	0.07	0.15
BiLSTM + FT^d^	88.08	89.48	87.17	0.21	0.07	0.08	0.03	0.09
BiLSTM + ELMo^e^	88.03	88.81	87.38	0.24	0.1	0.08	0.03	0.1
BiLSTM + RBS	83.74	88.46	80.24	0.27	0.08	0.13	0.03	0.09
BiLSTM + FT + RBS	88.18	91.73	85.54	0.21	0.07	0.09	0.01	0.07
BiLSTM + ELMo + RBS	89.86	90.83	89.17	0.19	0.09	0.05	0.03	0.08
BiLSTM-CRF^f^	70.12	79.04	65.57	0.53	0.11	0.26	0.11	0.21
BiLSTM-CRF + FT	87.16	88.58	86.41	0.25	0.09	0.08	0.03	0.12
BiLSTM-CRF + ELMo	88.66	87.95	89.44	0.23	0.11	0.06	0.02	0.11
BiLSTM-CRF + RBS	84.16	88.56	80.73	0.27	0.09	0.13	0.03	0.09
BiLSTM-CRF + FT + RBS	87.74	89.72	86.25	0.22	0.08	0.08	0.02	0.09
BiLSTM-CRF + ELMo + RBS	89.3	90.4	88.31	0.20	0.08	0.06	0.02	0.09

^a^Models are described according to their components; if neither ELMo nor FT is mentioned, then we used skip-gram embedding.

^b^RBS: rule-based system (ie, the outputs are added as extra features to the input of the deep learning module).

^c^BiLSTM: bidirectional long short term memory.

^d^FT: FastText embedding.

^e^ELMo: embedding for language model.

^f^CRF: conditional random field.

### Comparison by Type of Annotation

Table 4 summarizes the metrics of the different models by type of entities. The rule-based system alone had the lowest F-measure for every class due to a very low recall (medication class: 7.22), but it had the highest precision for all classes with the exception of medication name and duration. Associating the rule-based system to a bidirectional long short term memory increased medication name, medication class, dosage, and condition metrics (F-measures: 3.13, 3.12, 2.06, and 6.26, respectively) but decreased the F-measure for frequency, duration, and route (F-measures: –1, –3.38, and –2.66, respectively).

**Table 4:** Medication information predictions metrics results by models.

Label	RBS				BiLSTM + ELMo				BiLSTM + ELMo + RBS		
	F-measure	Precision	Recall	F-measure		Precision	Recall	F-measure		Precision	Recall
Medication name	90.31	96.46	84.89	92.2		93.79	90.67	95.33		95.33	95.33
Medication class	13.33	87.5	7.22	62.3		66.28	58.76	64.36		61.9	67.01
Dosage	90.43	96.62	84.98	92.17		91.13	93.23	95.29		95.52	95.05
Frequency	86.13	98.89	76.28	92.8		93.3	92.31	92.24		93.04	91.45
Duration	48.89	49.25	48.53	82.17		86.89	77.94	78.79		81.25	76.47
Route	47.92	85.19	33.33	75.52		72.97	78.26	72.86		71.83	73.91
Condition	33.64	100	20.22	55.9		62.5	50.56	62.16		77.97	51.69

^a^RBS: rule-based system

^b^BiLSTM: bidirectional long short term memory.

^c^ELMo: embedding for language models.

## Discussion

### Principal Findings

Our system achieved state-of-the-art performance for the task—an F-measure of 95.33 for medication names and an F-measure of 95.29 for dosage detection. Interestingly, these results were obtained using a data set representing only 10% of the size of similar data sets (N2C2 2018 shared task [22]). Combining expert knowledge (rule-based system) with a deep learning system increased the global F-measure, increased precision, increased recall, and decreased the slot error rate, having the most significant impacts on medication name, medication class, and dosage. While the rule-based system alone achieved the best precision and the worst recall, its association with the deep learning models helped to increase recall (for all information except condition) and increase precision (only for medication name, dosage, and condition of the intake). Adding a deep learning system with the embedding for language model on top of the rule-based system increased F-measures and recall for all categories. Adding a conditional random field layer increased the performance for the most frequent categories (ie, medication name, dosage, frequency). For other entities (ie, duration, route, condition), models with a conditional random field layer did not improve results (Multimedia Appendix 1). These results are consistent with those in the literature [18].

### Technical Significance

It is interesting to note that leveraging the synergy between expert knowledge and deep learning allowed us to achieve performance comparable to state-of-the-art with only 10% of the data. Infusing knowledge into deep neural networks will probably be a key element in the future progress of the field. The use of externally trained embeddings is a first step in this direction given that they allow the incorporation of latent knowledge from large corpora into the models. The impact of contextualized embeddings proves that a more accurate representation is even more important. We can expect improved performance with more recent language representation approaches such as BERT [49] or XLNET [50]; however, the cost for fitting these types of models, in terms of computation, time, and data, will be a challenge for languages other than English, for which resources (ie, data) are less available. Therefore, it will be valuable to leverage other types of representations (such as ontologies) to infuse knowledge into neural networks. A possible path could be through specific embedding techniques such as Poincaré embeddings [51].

Our approach is highly versatile. It can be transposed to any language, as long as writing expert rules is feasible. We used regular expressions to this end, but any rule based can be used. Our approach is also transposable to other information extraction use cases (or even text classification).

### Clinical Significance

The performance achieved by the system opens the way toward a large-scale use in real-life settings. We are currently developing an implementation to perform the medication information extraction at the scale of our institution. The versatility of the approach will enable its transposition to other types of clinical entities and information.

### Related Works

Compared with systems developed on the I2B2 2009 medication data set, the performance of our system is competitive [31]. Regarding token metrics, we showed better performance (medication name, dosage, frequencies, and duration token-level F-measures: +5.03, +4.49, +4.54, +28.89, respectively). However, a direct comparison is difficult given that the data sets are different. First, we trained and evaluated our models on a different corpus of French clinical notes. Also, because of language differences, the annotation guidelines were not strictly identical.

In our corpus, the vast majority of medication name slots contained only one token (48.7% of the medication names in the dictionary contain only one token), therefore, we can approximate a phrase-level F-measure using the token-level F-measure for medication names to compare with those in recent studies: Tao et al in 2018 reported a medication F-measure of 90.7 on the I2B2 corpus, and we achieved an F-measure of 95.3 [21]. However, regarding the mode of administration, our result was lower (token-level F-measures: 72.9 vs 93.3).

In French-language clinical data sets, mode of administration mentions are less structured and more variable than those in English-language clinical text. Therefore, it is logical to see lower results in this field, and our findings were consistent with the findings from a previous study [32]. Moreover, we took the condition of the intake, and not the reason for the intake, into consideration (which is more specific), and we added a tag regarding the class name; therefore, overall F-measures cannot be compared. Compared with results from a study [33] using a different French-language corpus that obtained a token-level F-measure of 90.4, our system’s raw results were higher. Comparisons should be made with caution because the corpus used in [33], though in the same language, was from a different source and contained only 147 documents.

The rule-based system offered the highest precision in most classes. The combination of deep learning and rule-based system could not maintain this high level of precision. One explanation could be that the performance of the rule-based system on the training set led the deep learning module to rely heavily on it. But when the rule-based system failed to generalize on the evaluation set, it caused a drop in accuracy in the hybrid system. This issue could be overcome by forcing the machine learning system to not exclusively rely on one source of information, contextual embedding or rule-based system features, by adding dropouts to the inputs.

Using a rule-based system associated with a deep learning model had two major benefits: the synergy between the rules and the machine learning increased the performance and the preannotation of the documents with the rules decreased the annotation time. Even if hybrid systems had already proved to be efficient [16,21,31,33,52], combining expert knowledge (rules) and latent knowledge (neural network), demonstrated a synergistic effect by increasing the performance in all metrics. It will be interesting to also evaluate approaches combining rules and deep learning in a reverse manner—first using a deep-learning model and refining the results using rules.

### Limitations and Perspectives

We have several perspectives from which to continue this work. First, we did not reproduce our study on a standard corpus such as that of the I2B2 challenge. We would, therefore, have to redevelop all the expert rules for this English corpus. Second, the embedding for language model was trained on a set of 100,000 French clinical notes from a single hospital [53]. However, even with these limits, using the embedding for language model proved to be efficient. We can anticipate even better results with an embedding for language model trained on a larger and more diverse corpus. Finally, our study focused on recognizing medication information entities without extracting the relationships among them. Tao et al [21] described a way to model the relationships by predicting boundaries of utterances that contain related medication entities. We plan to extend this to all types of sentences in our corpus, independently of the number of medications mentions. To this end, we will build a multitask model to predict medication fields and relations. We will also predict medication event markers such as start, stop, increase, decrease, switch, or unique intake of medication. Moreover, we could also predict meta-attribute markers that would provide information on the experiencer (patient, family, other), temporality (in the past, present, or for the future), and certainty (eg, factual, suggested, hypothetical, conditional, negated, or contraindicated [54]).

### Conclusion

The combination of expert rules, deep contextualized embedding (embedding for language model), and deep neural networks improved medication information extraction. This association achieved high performance on a heterogeneous corpus of French-language clinical reports, despite the data set’s small size.

## Appendix

Multimedia Appendix 1 Supplementary tables.

Multimedia Appendix 2 Formulas.

## Abbreviations

AP-HP: Assistance Publique–Hôpitaux de Paris
IOB: inside, outside, beginning
