# Supplementary material for: Hybrid Deep Learning for Medication-Related Information Extraction From Clinical Texts in French: MedExt Algorithm Development Study
Source: JMIR Med Inform. 2021 Mar 16;9(3):e17934. doi: 10.2196/17934 (PMC8077811; doi:10.2196/17934)
Supplement: Multimedia Appendix 1 [file medinform_v9i3e17934_app1.docx]

**Multimedia Appendix 2**

Table S1. Rules examples and regular expressions examples.

| **Tags** | **Rules** | **Regex** |
| --- | --- | --- |
|  |  |  |
| Dosage | NUMERIC VALUE with UNITS (mg, ui…) followed by end word or ’x’ | ([0-9]+,?[0-9]{03}\|[0-9]{13} ?[0-9]{03} ?[0-9]{03}\|,chiffre,) ?(,unite, \|\%)(?=\|x\|\.\|$) |
| Frequency | NUMERIC VALUE with FREQUENCY WORDS (par, /) and TIME WORDS (jour, semaine, heures) and possibly preceded by multiple markers (x, fois…) | (\|x\|fois)? ?([1-9] a )?([1-9]\|chiffre) ?(le)? ?(matin et soir\|matin,? midi et soir\|matin\|midi\|soir\|(au )?coucher\|chaque semaine\|chaque jour\|\/24h\|par 24h\|\/ ?jo*u*r*s?\|par semaine\|par jour\|\/ ?semaine\|\/ ?h) |
| Duration | NUMERIC VALUE with TIME WORDS preceded by duration words (pendant, pour…) | (?<=(pour\|pendant\|pdt\|sur) ?(les?)? ?)([0-9]\|number){13} ?(premiere?s?)? ?(jo*u*r*s*\|se*m*a*i*n*e*s*\|mo*i*s*\|ans*\|he*u*r*e*s*) |
| Route | ROUTE WORDS: intraveineux, iv, per os… | Route Lexicon |
| Condition | CONDITION WORDS with maximum 30 characters after medication name | (?<=drug.{030})(si\|en cas d?u?e?\|pour\|en raison d?u?e?) [a-z0-9, ]* |

Table S2. Models hyperparameters.^a^

|  | **Batch size** | **Dropout1** | **Dropout2** | **LSTM** **size** | **Recurrent Dropout** |
| --- | --- | --- | --- | --- | --- |
|  |  |  |  |  |  |
| BiLSTM | 64 | 0.7 | 0.2 | 512 | 0.1 |
| BiLSTM + RBS | 64 | 0.4 | 0.1 | 256 | 0.2 |
| BiLSTM + CRF | 128 | 0.2 | 0.1 | 512 | 0.6 |
| BiLSTM + RBS + CRF | 64 | 0.5 | 0.1 | 128 | 0.3 |
| BiLSTM + FT | 64 | 0.2 | 0.5 | 512 | 0.1 |
| BiLSTM + FT + RBS | 64 | 0.7 | 0.2 | 512 | 0.1 |
| BiLSTM + FT + CRF | 64 | 0.5 | 0.2 | 512 | 0.2 |
| BiLSTM + FT + RBS + CRF | 64 | 0.5 | 0.1 | 128 | 0.3 |
| BiLSTM + ELMO | 128 | 0.7 | 0.5 | 256 | 0.3 |
| BiLSTM + ELMO + RBS | 64 | 0.7 | 0.7 | 128 | 0.7 |
| BiLSTM + ELMO + CRF | 128 | 0.5 | 0.7 | 256 | 0.7 |
| BiLSTM + ELMO + RBS + CRF | 64 | 0.7 | 0.7 | 128 | 0.7 |

^a^ Models are described by their architecture components: BiLSTM = Bidirectional Long Short Term Memory, CRF = Conditional Random Field, ELMo = Embedding for language model, FT = FastText embedding, (if nor ELMo or FT is mentioned, then we use a skip gram embedding), RBS = Rule based System
