# Supplementary material for: Hybrid Deep Learning for Medication-Related Information Extraction From Clinical Texts in French: MedExt Algorithm Development Study
Source: JMIR Med Inform. 2021 Mar 16;9(3):e17934. doi: 10.2196/17934 (PMC8077811; doi:10.2196/17934)
Supplement: Multimedia Appendix 2 [file medinform_v9i3e17934_app2.docx]

**Multimedia Appendix 1**

$Recall=\frac{TP}{TP+FN}$

$Precision=\frac{TP}{TP+FP}$

$F1score=2*\frac{(Recall*Precision)}{(Recall+Precision)}$

$$SER=\frac{insertion+deletion+0.5*(type+frontier)}{Nslots}$$
